# The procedure for intraoperative graft verification in coronary surgery: Is high resolution epicardial imaging useful in addition to Transit-Time flow measurement to reduce postoperative failures?

**DOI:** 10.1186/1749-8090-10-S1-A279

**Published:** 2015-12-16

**Authors:** Gabriele Di Giammarco, Daniele Marinelli, Massimiliano Foschi, Tariq Mobariki, Donato Micucci, Francesca Faragalli, Michele Di Mauro

**Affiliations:** 1 Department of Cardiac Surgery, University "G. D'Annunzio" Chieti-Pescara, 66100, Chieti, Italy

## Background/Introduction

Transit-Time Flow Measurement (TTFM) is a valid method of intraoperative graft verification. TTFM shows a low diagnostic accuracy due to high number of false positive.

High-Resolution Epicardial Ultrasonography (HR-ECUS) is a well-recognized procedure for coronary anastomosis and stenosis evaluation with high sensitivity and specificity.

## Aims/Objectives

The aim of the study is to verify if the combined use of TTFM and HR-ECUS in intraoperative graft verification process can increase the diagnostic accuracy of procedure itself.

## Method

From November 2009 to December 2014, 741 patients underwent isolated CABG. A total number of 1733 grafts were performed; all the grafts were intraoperatively verified by means of both TTFM and HR-ECUS.

## Results

Among all grafts considered functioning at TTFM, 7 (0.4%) were failing at HR-ECUS and promptly redone. These were confirmed as true positive at graft revision due to technical error. HR-ECUS confirmed the good functioning of the remaining grafts already demonstrated by TTFM; among them, 8 showed high troponin I release (clinical false negative), whereas the remaining had no high TnI release (clinical true negative). In 2 of 39 grafts malfunctioning at TTFM, HR-ECUS confirmed the graft failure. Finally, in 35 cases, HR-ECUS did not confirm TTFM diagnosis demonstrating a full patency of the anastomosis; these grafts had an uneventful clinical course (true negative). The main result of this study is the increase of PPV from 10% with TTFM to almost 100% of TTFM + HR-ECUS, avoiding many unnecessary graft revisions.

## Discussion/Conclusion

In intraoperative graft verification procedure the combined use of TTFM and HR-ECUS increase diagnostic accuracy of the verification process close to 100%. Both methods should be used intraoperatively in order to achieve the best outcome for the patient.

